# Evaluating gait system vulnerabilities through PPO and GAN-generated adversarial attacks

**DOI:** 10.1038/s41598-026-37011-1

**Published:** 2026-01-23

**Authors:** El Mehdi Saoudi, Jaafar Jaafari, Said Jai Andaloussi

**Affiliations:** Laboratory of Data Engineering and Intelligent Systems, Faculty of Sciences Ain Chock, Hassan II University of Casablanca, Casablanca, 20000 Morocco

**Keywords:** Adversarial Machine Learning, Reinforcement Learning Security, Deep Learning Vulnerability, Gait Analysis Technology, Biometric System Analysis, Computational science, Computer science

## Abstract

This study delves into the vulnerabilities of deep learning-based gait recognition systems against adversarial attacks, a critical issue considering the increasing reliance on these technologies in high-security environments. We highlight a major issue concerning the susceptibility of these systems to adversarial interventions that compromise their reliability. The importance of this issue stems from the critical role of gait recognition in applications where security and accuracy are paramount. Our approach introduces an advanced methodology that integrates Proximal Policy Optimization (PPO) with Generative Adversarial Networks (GANs) to create and deploy adversarial attacks in the form of targeted adversarial patches. These patches are designed to deceive gait recognition algorithms without detection by human oversight, exploiting the models’ weaknesses to induce misclassification. This methodology not only leverages the strengths of GANs to produce deceptive examples but also innovatively utilizes PPO to ascertain their optimal placements, thereby maximizing the disruption on gait recognition systems. We assess the impact of these attacks using the CASIA Gait Database: Dataset B and the OU-ISIR Treadmill Dataset B - Clothes variation-, covering both real-world and controlled environments. Our results demonstrate a significant decline in recognition accuracy post-attack, underscoring the effectiveness of our adversarial approach. These findings underscore critical security flaws and actively inform the broader discussion aimed at boosting the robustness of gait recognition systems. The impact of our research extends significantly, providing crucial insights that aid in the creation of more secure, attack-resistant biometric recognition systems, thereby enhancing the resilience of gait recognition technologies against the backdrop of advancing cyber threats.

## Introduction

The advent of the 21st century has seen significant advances in the field of technology, particularly in artificial intelligence (AI), machine learning (ML), and deep learning (DL). These advances have radically transformed numerous sectors, introducing automation and improved decision making capabilities^1,2^. Among these developments, gait recognition has emerged as a prominent area, leveraging unique walking patterns to identify individuals. Unlike traditional biometric techniques, gait recognition offers a non-invasive approach, relying on motion analysis rather than direct physical contact or high-resolution images. This characteristic makes it particularly suitable for various applications, including surveillance, forensic investigations, and healthcare^3–5^.

Deep learning, especially through Convolutional Neural Networks (CNNs) and Recurrent Neural Networks (RNNs), has been a game-changer in gait recognition. These algorithms excel in extracting complex patterns from extensive datasets, enabling models to learn from experiences and make informed decisions. The integration of deep learning into gait analysis has opened new avenues for accurate and reliable identification, marking a significant leap in intelligent self-learning systems^5^. However, the implementation of deep learning techniques in gait recognition systems has revealed a significant challenge, which is the vulnerability to adversarial attacks. These attacks involve crafty alterations to the input data that are designed to mislead AI systems, leading to incorrect classifications or predictions^6,7^. Such adversarial threats are a matter of grave concern, especially in applications where the reliability and security of gait recognition are paramount. Addressing these vulnerabilities is crucial for ensuring the robustness and reliability of gait recognition technologies in various scenarios, from surveillance to access control systems^8^.

To investigate these concerns, this study adopts an evaluation strategy based on the use of Generative Adversarial Networks (GANs)^11^ and Proximal Policy Optimization (PPO)^9,10^ as tools to assess and test the robustness of deep learning-based gait recognition systems. The goal is not to develop new attack techniques but rather to leverage GANs-known for their ability to generate deceptive adversarial examples-and PPO-recognized for its reinforcement learning capabilities-to systematically expose weaknesses in the target models. GANs are used to generate adversarial patches, while PPO is employed to optimize their placement for maximum impact. This combined approach enables us to simulate sophisticated attacks and examine the system’s response under controlled adversarial conditions.

Recognizing the urgency to counter these vulnerabilities, this paper focuses on evaluating and quantifying the robustness of gait recognition systems when exposed to adversarial interventions. Our methodology utilizes adversarial patches generated by GANs and strategically placed using PPO to simulate realistic and targeted attacks. This evaluation framework aims to provide a deeper understanding of the limitations and resilience of current gait recognition models.

This study uniquely applies PPO to optimize the placement of GAN-generated adversarial patches in the context of gait recognition-a novel application of reinforcement learning in this domain. While GANs have been previously used to produce adversarial examples, the originality of our work lies in employing PPO to determine optimal spatial locations for those patches, thereby intensifying their disruptive potential. The contribution of our research is thus twofold: (1) demonstrating how a combined PPO-GAN approach can be used to simulate realistic threats, and (2) providing a rigorous assessment of the vulnerabilities present in state-of-the-art gait recognition systems.

The remainder of this paper is organized as follows: Section 2 presents a review of relevant literature, focusing on deep learning applications in human gait recognition and the implications of adversarial attacks. Section 3 details our proposed methodology, including the creation of adversarial patches using PPO and GANs, and their application in evaluating the robustness of gait recognition systems. Sections 4 describe the experimental setup, datasets, evaluation metrics, and discuss the results, offering a comparative analysis with existing methods. Section 5 concludes the paper by summarizing key findings, discussing potential limitations, and suggesting directions for future research to enhance the resilience of gait recognition systems against adversarial attacks.

## Related works

In the evolving landscape of biometric recognition, human gait analysis has emerged as a pivotal area of research, primarily due to its non-invasiveness and difficulty in obfuscation human gait analysis has emerged as a pivotal area of research, primarily due to its non-invasiveness and the inherent difficulty for individuals to consciously alter or disguise their gait in real-world environments. The advancement of deep learning methodologies has further propelled this field into new frontiers, offering sophisticated approaches for capturing and interpreting the subtle nuances of human gait. This section explores into the existing body of literature, dissecting two fundamental aspects that are crucial to the understanding and advancement of gait recognition technology.

Initially, we explore the impact of deep learning applications in human gait recognition, tracing the evolution of this technology from its nascent stages to its current state-of-the-art incarnations. This exploration includes an examination of the various deep learning models and algorithms that have significantly enhanced the accuracy and efficiency of gait recognition systems. Subsequently, we shift our focus to the realm of adversarial attacks, a burgeoning concern in the field of artificial intelligence, particularly in the context of biometric recognition systems. We examine the various forms of adversarial attacks that threaten the integrity of gait recognition systems and survey the existing literature on the strategies devised to mitigate these risks. This comprehensive review sets the stage for our study, framing the current challenges and illuminating the paths for future research endeavors in enhancing the robustness of gait recognition systems.

The incorporation of deep learning in human gait recognition has catalyzed a revolutionary shift in the field, enhancing the identification of distinct walking patterns through advanced data processing and pattern recognition capabilities. Studies in this realm have substantially employed deep learning to refine gait recognition processes, spanning a range of methodologies from neural network architectures to performance improvement strategies under varied conditions^4,5,12^.

Deep learning-based gait recognition methods have evolved from traditional approaches using handcrafted features^13–17^ to sophisticated neural network models that autonomously extract high-level features from raw gait data. This transition highlights a significant shift from conventional techniques like template-based methods to more advanced architectures such as Convolutional Neural Networks (CNNs) and Recurrent Neural Networks (RNNs)^18–22^. The efficiency and adaptability of these models allow them to handle the intricate spatial-temporal dynamics inherent in gait data, offering a robust solution for accurate gait recognition.

Recent research has introduced innovative deep learning strategies to further enhance gait recognition accuracy. In Table 1, we offer a comprehensive overview of recent studies in the field.

**Table 1:** Summary of Recent Research in Deep Learning-Based Gait Recognition.

References	Method	Main contribution/focus	Outcomes	Shortcomings
Tran et al.^23^	LSTM networks combined with CNNs	Improved recognition using Inertial Measurement Units.	High accuracy in diverse conditions	Requires large dataset for training, potentially limiting rapid deployment.
Liu et al.^24^	CNN-LSTM network	Optimization of gait recognition using 3D skeletal information.	Enhanced spatial-temporal feature integration	Computationally intensive, may not be optimal for real-time applications.
Amin et al.^25^	Conv-BiLSTM and YOLOv2-squeezeNet models	Enhanced recognition with notable prediction scores.	Improved prediction accuracy across multiple datasets	Complexity of model architecture increases the computational resources required.
Amanulla et al.^26^	Clustering-based Faster RCNN	Classifying gait into suspicious and non-suspicious patterns.	Effective classification with high accuracy	Limited to specific walking patterns, may not generalize well to all scenarios.
Mehmood et al.^27^	Densenet-201 with a hybrid feature selection	Resilience to covariates like clothing and items carried.	Robust performance against various covariates	Fine-tuning required for optimal feature selection, which can be time-consuming.
Sepas-Moghaddam et al.^3^	comprehensive overview of breakthroughs and recent developments in gait recognition with deep learning.	cover broad topics including datasets, test protocols, state-of-the-art solutions, challenges, and future research directions.	Provides a broad perspective on the field	May not provide the in-depth technical detail needed for all reader groups.

The continued exploration and development in this domain underscore the vast potential of deep learning in enhancing gait recognition systems. These advancements not only address existing challenges but also pave the way for more resilient and efficient gait recognition technologies. As the field progresses, it becomes increasingly evident that the integration of sophisticated deep learning techniques is indispensable for advancing gait recognition capabilities, particularly in the face of complex real-world scenarios and the ever-evolving landscape of adversarial attacks. Each of these studies contributes significantly to the body of knowledge in gait recognition, offering diverse perspectives and methodologies that collectively enhance the understanding and application of deep learning in this field.

As the field of human gait recognition continues to evolve with the integration of deep learning techniques, the threat landscape has expanded, prominently featuring adversarial attacks. These sophisticated and often subtle intrusions into the data realm pose a serious challenge to the integrity and reliability of gait recognition systems, particularly in sensitive areas such as security and surveillance. This subsection transitions from discussing the deep learning applications in human gait recognition to addressing the imminent threat of adversarial attacks in this domain.

Adversarial attacks in the context of gait recognition leverage designed perturbations to input data, with the intent to deceive deep learning models into erroneous classifications. These perturbations, often imperceptible to the human eye, can significantly distort the model’s output, resulting in substantial inaccuracies. The implications of such threats are particularly severe in areas where the precision and dependability of gait recognition are paramount^28–30^.

Two primary types of adversarial attacks prevalent in this realm are white-box^31^ and black-box attacks^32^. White-box attacks operate with full knowledge of the target model, including its architecture and parameters, and use this information to create bespoke adversarial input. In contrast, black-box attacks are executed with limited or no prior knowledge of the internal structure of the model, often relying on a trial-and-error approach or substitute models to generate effective adversarial input^29^. Additionally, these attacks are further classified as targeted or non-targeted, with the former focusing on manipulating the model to misclassify input into a specific incorrect class, and the latter aimed at causing any form of misclassification.

The rise of these complex attack vectors has necessitated the development of robust deep learning models and the formulation of methods to detect and counteract such threats, thereby ensuring the continued efficacy and security of gait recognition systems. The remainder of this subsection highlights recent research that has delved into the realm of adversarial attacks within the context of human gait recognition, employing deep learning techniques.

Table 2 provides a comprehensive overview of recent studies that focus on the impact of adversarial attacks on gait recognition. These studies collectively underscore the critical need for enhanced security measures in gait recognition systems. As adversarial attacks continue to evolve in sophistication, the importance of developing more resilient and robust deep learning models capable of withstanding these threats becomes increasingly paramount. Acknowledging the susceptibility of human gait recognition systems to adversarial threats, our research shifts focus towards assessing their resilience. In the “Proposed Method” section, we present a novel adversarial approach using Proximal Policy Optimization and Generative Adversarial Networks. This methodology is centered on crafting and implementing adversarial patches to challenge and evaluate the robustness of deep learning-based gait recognition models. This critical part of our study is dedicated to exposing potential vulnerabilities in these systems, offering valuable insights for future advancements in enhancing their security against evolving adversarial tactics.

**Table 2:** Summary of Research on Adversarial Attacks in Gait Recognition and Other Fields.

References	Method	Main contribution/focus	Outcomes	Shortcomings
He et al.^33^	Temporal sparse adversarial attack using GAN	Introduce high-quality adversarial gait silhouettes to diminish model accuracy.	Demonstrates effective attack while maintaining imperceptibility.	Requires complex generation and insertion techniques.
Li et al.^34^	Semi-white-box adversarial attack using GANs	Generate adversarial examples disturbing gait model output.	Emphasizes the need for advanced adversarial defense.	Limited to specific model architectures and settings.
Maqsood et al.^35^	Single-pixel adversarial noise manipulation	Subtle yet impactful attacks on GEIs demonstrating the necessity for robust defenses.	Shows significant effects with minimal perturbation.	May not generalize across different models or conditions.
Chen et al.^36^	Anti-forensic framework using GANs	Manipulate camera model traces to deceive CNN-based classifiers.	Effective in various adversarial scenarios.	Focused on image forensics, extrapolation to gait recognition is hypothetical.

In line with our investigation of adversarial threats to biometric systems, several recent GAN-based contributions deserve mention. Antil and Dhiman^37^ proposed a GAN-powered architecture for face anti-spoofing, integrating MSRCR and CBAM modules to improve generalization across lighting and background conditions. Although focused on facial recognition, their use of GANs for robustness assessment highlights parallels with our approach in gait-based systems. Hu et al.^38^ introduced the Discriminant Gait GAN (DiGGAN), which enhances cross-view gait recognition by generating view-transferred gait images as visual evidence, demonstrating GANs’ effectiveness in handling covariates like camera angle. While their goal differs, the application of GANs to gait data aligns with our interest in their generative capacity. Finally, Ren et al.^39^ proposed GanFinger, a GAN-based fingerprinting method for neural network ownership verification using adversarial examples. Although their focus is on intellectual property protection, their technique of generating imperceptible adversarial examples to expose model behavior is conceptually relevant to our use of adversarial patches for evaluating system resilience.

## Proposed method

At the onset of our proposed methodology, it is crucial to delve into the specifics of the adversarial attacks we address in this study. Adversarial attacks in the context of deep learning-based gait recognition systems are intricately designed to subtly manipulate the input data, targeting the model’s latent vulnerabilities without conspicuously altering the apparent gait pattern. In our research, the adversarial attacks are not random distortions but are meticulously calculated using Proximal Policy Optimization (PPO) combined with Generative Adversarial Networks (GANs) to create and optimally position adversarial patches. These patches are integrated into the input data, maintaining their visual semblance to genuine gait patterns to avoid detection by human observers while ensuring they are interpreted erroneously by the system.

Our proposed methodology, is primarily aimed at identifying potential vulnerabilities within the gait recognition system by simulating adversarial attacks. This process is instrumental in understanding how such attacks could compromise the system. The insights gained from this evaluation are crucial for developing and implementing strategies to mitigate these vulnerabilities, thereby enhancing the system’s resilience against potential adversarial threats. By identifying the specific points of failure, our method aids in identifying where the system requires reinforcement, guiding the development of targeted countermeasures to strengthen the system’s defenses.

Employing PPO allows us to make informed decisions about the placement of these adversarial patches, optimizing their location and intensity to exploit specific weaknesses within the gait recognition system. The goal is to induce a classification error, misleading the system into either misidentifying or failing to recognize the altered gait pattern, which, though subtly modified, retains essential characteristics of the original. This strategic manipulation capitalizes on the ’blind spots’ within the system’s learning algorithm areas where the model is particularly sensitive to input perturbations, yet these vulnerabilities are not apparent through standard validation processes.

By initiating our methodology section with this detailed account, we set the stage for a comprehensive understanding of the adversarial framework we propose. This framework underscores the novelty and specificity of our approach, leveraging the nuanced interplay between PPO and GANs to craft attacks that challenge the model’s integrity at its most susceptible points.

We initiate this section by providing a detailed overview of the datasets that form the basis of our experimental evaluation, emphasizing their relevance and diversity in mirroring real-world scenarios. Following this, we introduce our data representation technique, which is pivotal in distilling and preparing gait data for effective analysis. The subsequent sections unfold our adversarial strategy: from the innovative generation of adversarial patches using GANs, designed to disrupt the recognition process, to the precise deployment of these patches within Gait Energy Images (GEIs) using the PPO algorithm. This combination of GANs and PPO exemplifies a novel approach in adversarial attacks, specifically designed to challenge and reveal potential weaknesses in gait recognition models.

### Dataset selection

**Fig. 1:**
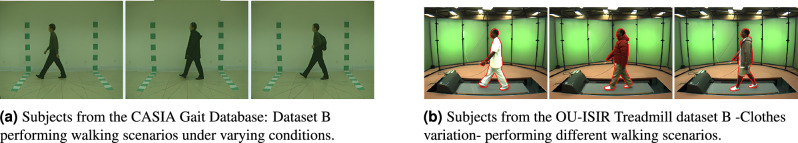
Visual representation of the CASIA and OU-ISIR gait datasets.

To comprehensively assess the efficacy of our proposed method, we selected two prominent datasets: the CASIA Gait Database: Dataset B^40^ and the OU-ISIR Treadmill dataset B -Clothes variation-^41^. The rich diversity and complexity presented by these datasets render them ideal for evaluating the robustness and performance of our gait recognition approach. The CASIA Gait Database: Dataset B, curated by the Chinese Academy of Sciences, is a multiview gait database renowned for its extensive collection of video sequences showcasing various walking scenarios (Fig. 1a). This dataset encompasses 124 subjects, each represented by 10 walking sequences across 11 camera angles, including scenarios where individuals walk normally, carry bags, and wear different types of coats.

On the other hand, the OU-ISIR Treadmill dataset B -Clothes variation-, assembled by the Institute of Intelligent Systems and Robotics, offers a controlled experimental setting with uniform lighting, consistent camera positioning, and standardized backgrounds. This dataset is composed of gait silhouette sequences of 68 subjects from a side view with clothes variation, featuring up to 32 combinations (Fig. 1b). Such a controlled environment facilitates a detailed and comparative analysis of gait recognition algorithms, allowing for rigorous evaluation against a wide range of clothing conditions.

The strategic use of both datasets, each characterized by their respective real-world and controlled experimental conditions, establishes a robust framework for the thorough evaluation and validation of our method’s efficacy. This dual-dataset approach ensures a comprehensive exploration of the proposed method’s performance, enhancing its generalization capabilities across a variety of gait recognition scenarios.

All methods were carried out in accordance with relevant guidelines and regulations. All experimental protocols for the CASIA Gait Database: Dataset B were approved by the Institute of Automation, Chinese Academy of Sciences Ethics Committee. For the OU-ISIR Treadmill dataset B -Clothes variation-, protocols were approved by the Ethical Committee of the Institute of Scientific and Industrial Research, Osaka University. Informed consent was obtained from all subjects and/or their legal guardian(s) as per the dataset’s original documentation.

### Gait data representation and processing

Efficient and meaningful processing of gait data is essential for our adversarial attack methodology. We utilized Gait Energy Images (GEIs) to represent gait data from the CASIA-B and OU-ISIR datasets due to their computational efficiency and effectiveness in capturing the nuances of gait patterns^42^. GEIs provide a compact representation by averaging silhouette images over a gait cycle, thereby preserving crucial spatiotemporal characteristics of the gait while reducing computational complexity.

#### Computation of GEI

The Gait Energy Image (GEI) for each pixel location $(x, y)$ in a gait silhouette frame $I$ is calculated by averaging all frames $t$ across the gait cycle $T$. This process is mathematically represented in Equation 1:

1 $$\begin{aligned} GEI(x, y) = \frac{1}{T} \sum _{t=1}^{T} I(x, y, t) \end{aligned}$$

By averaging the frames over the gait cycle, the GEI effectively consolidates critical spatiotemporal gait information into a single composite image. This approach streamlines the feature extraction process, which is crucial for the success of our adversarial attack strategy.

#### Data preparation and normalization

For the CASIA-B dataset, we first extracted human silhouettes from sequences using advanced silhouette extraction techniques^43^. We then normalized the size and aligned these silhouettes horizontally for consistency. Gait cycle segmentation involved determining the gait frequency and applying a maximum entropy estimation technique^44^. In contrast, the OU-ISIR dataset already provided pre-processed GEIs, which we adjusted to match our uniform dimension of $240 \times 240 \times 1$. This step ensured a consistent input data format across both datasets.

#### GEI differentiation

GEIs inherently contain both static and dynamic regions. Static regions provide structural information, while dynamic regions, more crucial for gait recognition, reflect the motion involved in gait. We focused on dynamic areas to minimize the impact of covariates such as clothing and carried objects, ensuring a more reliable identification process.

#### Dataset uniformity

Ensuring uniformity across datasets was vital for our methodological approach. For CASIA-B, after computing GEIs, we resized them to our standard dimension, and for OU-ISIR, we verified and adjusted the pre-prepared GEIs. This harmonization was essential for effective training and optimal performance of our adversarial models.

**Fig. 2:**
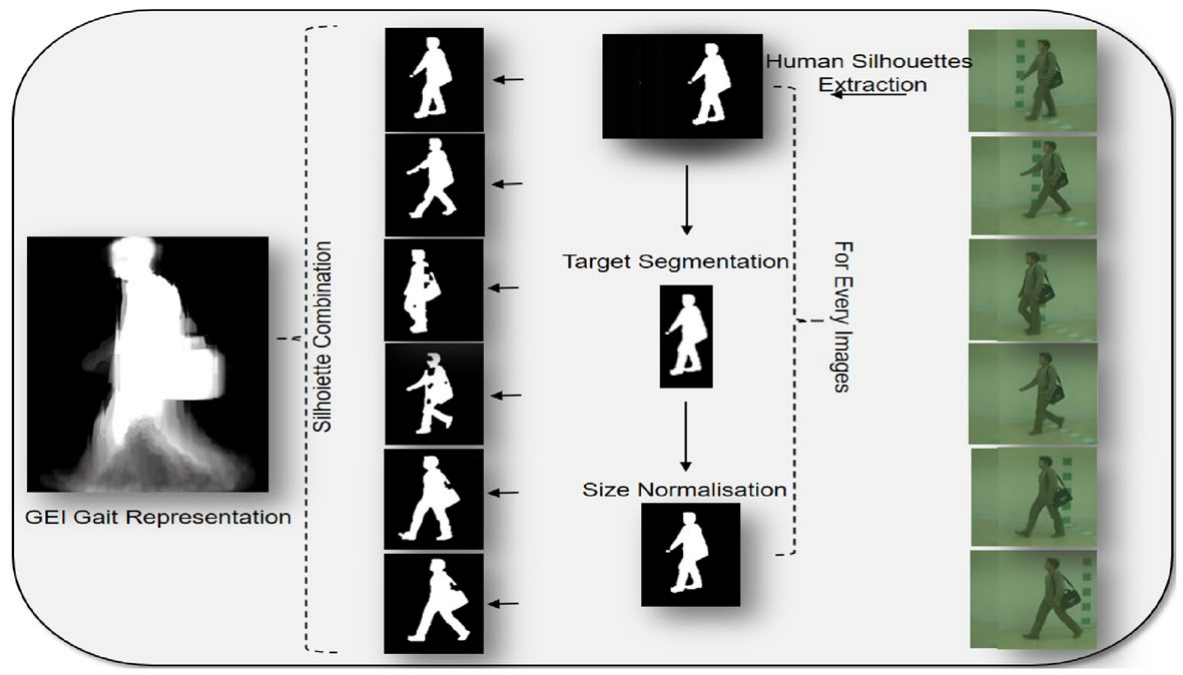
Workflow for Generating Gait Energy Images (GEIs) from raw gait sequences, highlighting the steps of human silhouette extraction, target segmentation, size normalization, and silhouette combination.

The entire process of computing and standardizing the GEI for a gait cycle, covering both the CASIA-B and OU-ISIR datasets, is depicted in Fig. 2.

### Proposed deep learning-based gait recognition model

In our research, we begin by developing a deep learning-based gait recognition model that serves as a critical component for evaluating the effectiveness of our adversarial patches. This model, crucial for our adversarial attack simulations, is a modified version of the state-of-the-art Convolutional Neural Network (CNN) architecture. Drawing inspiration from the framework proposed by Bukhari et al.^45^, our model incorporates essential adaptations to improve its ability to discern complex gait patterns.

**Fig. 3:**
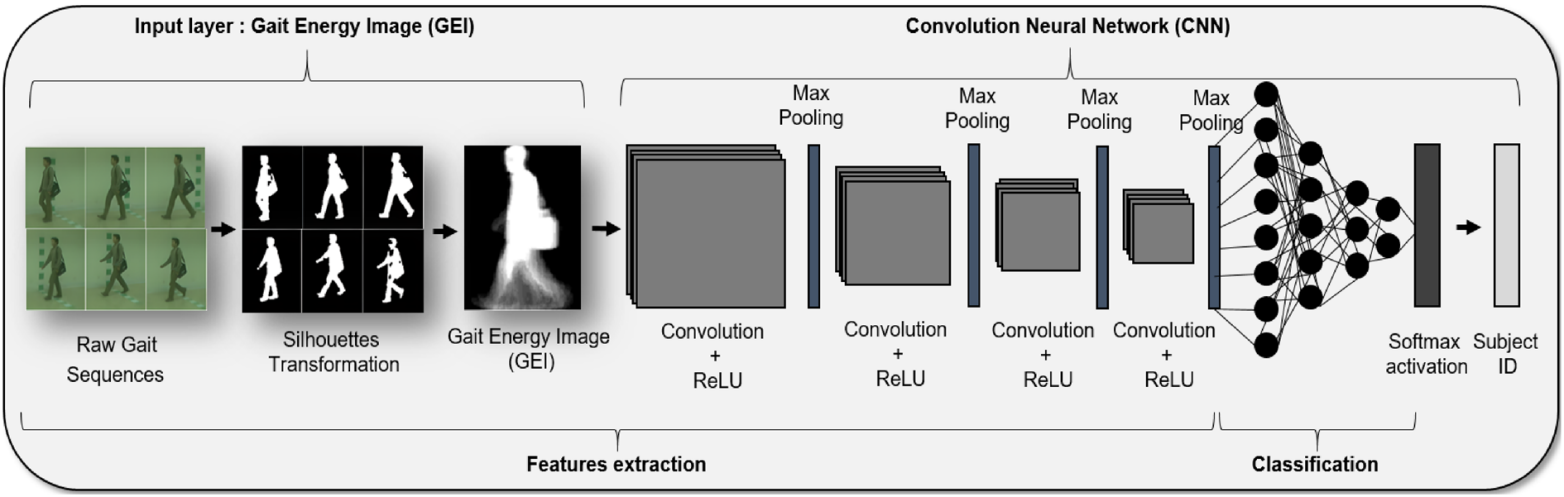
Architecture of the proposed deep learning-based gait recognition model. This diagram delineates the multi-layered approach employed to extract and learn discriminative features from Gait Energy Images (GEIs), essential for precise individual identification. The illustrated CNN model is foundational for our experiments, serving as the pivotal component upon which our adversarial attack simulations are constructed and evaluated, underscoring its integral role in validating the efficacy of our proposed adversarial strategies.

This CNN model serves as the basis for our experiments, wherein its resistance to adversarial manipulations is thoroughly examined. Specially designed to process Gait Energy Images (GEIs), the model is adept at extracting distinctive gait features for individual identification. Our enhancements focus on augmenting the model’s sensitivity to nuanced features in gait patterns, making it a realistic subject for adversarial attacks. The architecture of the model is detailed in Fig. 3.

Our CNN model, specifically designed for enhanced gait recognition, consists of 14 layers: six convolutional layers, six pooling layers, and two fully connected layers. This detailed layer structure, expanding beyond the traditional four convolutional layers often seen in similar models, allows for deeper analysis and processing of gait data. The model inputs a grayscale Gait Energy Image (GEI) measuring 240x240x1. The first four convolutional layers feature a 3x3 kernel size without padding. These layers are designed to generate 16, 32, 64, and 128 feature maps, respectively, providing a comprehensive analysis of the gait features captured in the GEI.

An essential improvement in our Convolutional Neural Network (CNN) model is the optimization of the Leaky Rectified Linear Unit (ReLU) activation function. Unlike standard configurations, we have adjusted the activation function with a customized negative slope factor of 0.05. This particular adaptation is vital in enhancing the model’s ability to discern finer details within Gait Energy Image (GEI) data, facilitating a more comprehensive capture of gait characteristics. The activation slope factor for the LeakyReLU function was empirically set to 0.05 after extensive experimentation. Several values were tested, including 0.01, 0.1, and 0.2. The value of 0.05 was found to offer the most stable convergence during training while enhancing the model’s ability to detect subtle variations in gait silhouettes. This fine-tuning allowed the network to better capture discriminative features in Gait Energy Images, leading to improved classification performance. The implementation of this distinct modification signifies a deliberate deviation from conventional methodologies, underscoring our dedication to advancing model performance. To further enhance our model’s architecture, each convolutional layer is paired with a 2x2 max pooling layer. This arrangement is carefully crafted to achieve an optimal balance between preserving essential gait information and reducing computational demands. The pooling layers play a significant role in diminishing the spatial dimensions of the feature maps, thereby contributing to the model’s overall efficiency.

**Fig. 4:**
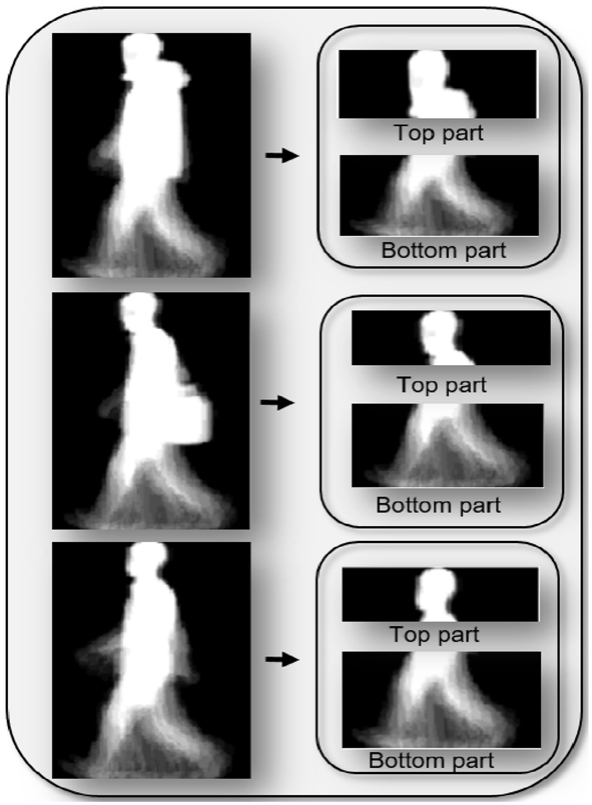
Visualization of the regions of interest (ROI) extraction process from gait energy images (GEIs).

To counter the potential interference of covariates such as bags and coats in gait recognition, our CNN model employs the concept of Regions of Interest (ROIs). These covariates, while common in real-world scenarios, can introduce noise into gait data, adversely affecting recognition accuracy. To circumvent this issue, we focus on ROIs within the Gait Energy Images (GEIs). These ROIs are specific areas within the images that are less affected by variations in clothing or carrying objects. The process of identifying these ROIs is grounded in the understanding that the core, discriminative gait features are predominantly located in regions less prone to alteration by external covariates. By honing in on these areas, our model is better equipped to concentrate on the intrinsic gait patterns that are consistent, regardless of the subject’s attire or carried items. This approach not only enhances the robustness of the recognition process but also ensures that our model remains focused on the most relevant aspects of the gait data for reliable identification. Figure 4 illustrates the process of extracting these Regions of Interest from the GEI images, showcasing our method’s focus on the most stable and informative segments of the gait data.

In conclusion, the proposed CNN model, depicted in Fig. 3, incorporates significant enhancements in terms of layer complexity, feature map generation, activation functions, and pooling strategies. These improvements are geared towards achieving a more efficient, precise, and robust system for gait-based individual identification. This CNN model lays the foundation for the subsequent phase of our research, where we delve into the intricacies of adversarial attacks.

### Enhanced GAN architecture for adversarial patch generation

Within this section, we detail the design of our Generative Adversarial Network (GAN) architecture, developed with a focus on generating adversarial patches. The primary aim of these patches is to probe and reveal the vulnerabilities of our developed deep learning-based gait recognition system to adversarial attacks. This enhanced GAN architecture is a critical component of our methodology, facilitating the creation of adversarial inputs that simulate potential security breaches in gait recognition applications.

**Fig. 5:**
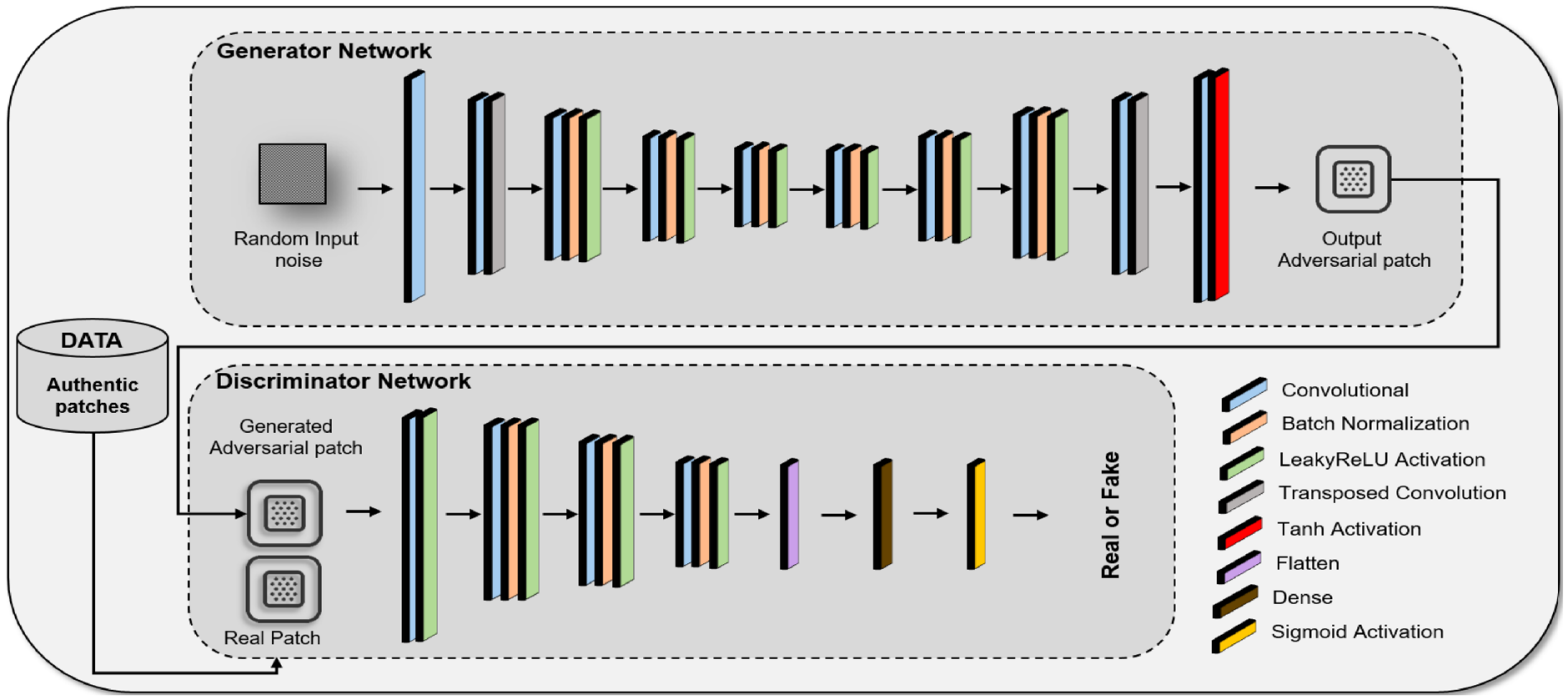
Architecture of the Generative Adversarial Network (GAN) for Adversarial Patch Generation. This schematic portrays the dual-network system comprising the Generator Network, which takes random noise as input and produces adversarial patches, and the Discriminator Network, which discerns between authentic patches and the synthetic outputs of the generator. The interplay between these networks results in the creation of adversarial patches finely tuned to disrupt the deep learning-based gait recognition models, without being discernible to human inspectors. This process is vital for generating the stealthy and effective adversarial patches used in testing the resilience of gait recognition systems within our experiments.

In general, a GAN consists of two primary components: the generator network, *G*, and the discriminator network, *D*. The generator is responsible for creating the adversarial patches, while the discriminator evaluates them against real data to ensure their efficacy and realism. The architecture of our GAN, illustrating the intricate interplay between *G* and *D*, is depicted in Fig. 5. Below, we delve into the specifics of our customized generator network.

#### Customized generator for adversarial patch

The proposed generator network, *G*, extends the standard GAN architecture with advanced features and layers that are specialized for generating gait-related adversarial imagery. Our network’s architecture is configured as follows:

##### Input layer

The generator begins with an input layer that accepts a noise vector *z*, sampled from a Gaussian distribution. This vector serves as the seed for generating adversarial patches.

##### **Convolutional layers**

In our generator network, *G*, the input layer is followed by a series of enhanced convolutional layers. These layers are configured with specific filter sizes and strides, each finely tuned to effectively capture and synthesize the unique characteristics of human gait.

- The first convolutional layer utilizes filters of size $3 \times 3$, with a stride of 1 and padding to maintain the spatial dimensions. This layer aims to capture the initial high-level features from the noise vector.
- Subsequent layers gradually increase in complexity. For instance, the second layer employs $5 \times 5$ filters with a stride of 2, delving deeper into the feature space to extract more detailed aspects of gait patterns.
- As the network progresses, the filter sizes and strides are adjusted to ensure a comprehensive analysis of the features relevant to human gait. This includes varying the filter dimensions and strides across different layers to target various aspects of the gait cycle.

Each convolutional layer in our generator network is followed by a batch normalization layer and then a LeakyReLU activation layer. The batch normalization layers are crucial in normalizing the outputs of the convolutional layers, ensuring consistent mean and variance. This not only stabilizes the training process but also promotes faster convergence by addressing internal covariate shift. Following batch normalization, the LeakyReLU activation function introduces non-linearity to the network, enabling it to learn complex patterns more effectively. The inclusion of LeakyReLU helps in avoiding vanishing gradient problems and allows the network to maintain the gradients flowing through the architecture. This deliberate configuration of convolutional layers, batch normalization, and LeakyReLU activation empowers our generator to produce high-fidelity adversarial patches that are both effective and subtle, enhancing the robustness of our adversarial attack methodology.

##### Output layer

The culmination of our generator network *G* is marked by its output layer, which plays a pivotal role in finalizing the structure of our adversarial patches. This layer is engineered to employ a hyperbolic tangent (tanh) activation function, which serves two critical functions:

- Normalization of Patch Intensities: The tanh activation function effectively scales the output of the generator, ensuring that the pixel intensities of the adversarial patches are normalized within a specific range. This normalization process is crucial as it transforms the output values to a range between -1 and 1.
- Enhanced Adversarial Effectiveness: By using the tanh function, we ensure that the adversarial patches generated possess a balanced contrast, improving their ability to manipulate the gait recognition model without being overtly noticeable to the human eye.

The generator’s functionality can be encapsulated in the following equation :

2 $$\begin{aligned} G(z; \theta _g) = \tanh (\text {Conv}^{(n)}(...\text {LeakyReLU}(\text {BatchNorm}(\text {Conv}^{(1)}(z; \theta _{g1})); \theta _{g2})...); \theta _{gn}) \end{aligned}$$

Where, *z* represents the input noise vector. $\theta _g$ denotes the collective parameters of the generator. $\text {Conv}^{(n)}$ symbolizes the nth convolutional layer in the sequence, and the intermediate stages involve LeakyReLU activation and batch normalization, indicating the complexity and depth of the network’s processing capabilities.

#### Discriminator network for patch validation

In our adversarial attack architecture, the discriminator network *D* plays an instrumental role in validating the effectiveness of the generated adversarial patches. This convolutional neural network is specifically calibrated to scrutinize and distinguish between authentic gait patterns and synthetic adversarial artifacts generated by *G*. The key components of our discriminator network *D* include:

##### Input layer

The input layer of the discriminator, *D*, is specifically designed to receive adversarial patches generated by the generator, *G*. These patches, which are essentially small image segments, are crafted to match the size and dimensionality of regions within genuine gait data images. This configuration ensures that *D* evaluates the patches in a manner consistent with how they would appear when applied to actual gait data, thereby facilitating a realistic and effective assessment. The discriminator’s task is to determine whether these patches, when placed within a gait image, would be effective in deceiving the gait recognition system.

##### Discriminative convolutional layers

In the discriminator network of our GAN, we implement a fixed architecture consisting of seven convolutional layers, each carefully designed to capture and analyze a diverse range of features. This configuration ensures the effective differentiation between authentic gait data and adversarial patches created by the generator, *G*. The first convolutional layer employs larger 7x7 filters with 32 channels, suitable for initial broad feature extraction. Subsequent layers use progressively smaller filters (5x5 in the second layer, followed by 3x3 filters in the remaining layers), with an increasing number of channels (64, 128, 256, 512, and so on). This design facilitates a gradual and deep analysis of the input data, ensuring that even the most subtle adversarial attributes are detected. After the convolutional layers, the network culminates in a densely connected layer with a sigmoid activation function, outputting a probability score to assess the authenticity of the input. This structured approach allows for a thorough and efficient evaluation of the adversarial patches’ effectiveness against our deep learning-based gait recognition model.

##### Output layer

The discriminator concludes with a sigmoid activation function, producing a probability score indicating the authenticity of the input data.

The discriminator’s decision-making process is captured by:

3 $$\begin{aligned} D(x; \theta _d) = \sigma (\text {Conv}^{(m)}(...\text {ReLU}(\text {Conv}^{(1)}(x; \theta _{d1})); \theta _{dm})) \end{aligned}$$

where *m* denotes the number of discriminative layers, and $\theta _d$ encapsulates the discriminator’s parameters.

#### Adversarial patch training procedure

Although pretrained layers are often used in GANs, we opted to train the network from scratch due to the domain-specific nature of gait silhouettes. Pre-trained models based on natural images (e.g., ImageNet) may fail to capture relevant features of Gait Energy Images. The combined diversity of the CASIA-B and OU-ISIR datasets was sufficient to support effective GAN training from scratch.

To effectively assess the robustness of deep learning-based gait recognition systems, the training of the Generative Adversarial Network (GAN) is pivotal. In our study, the generator (*G*) and discriminator (*D*) engage in a strategic min-max game, where they iteratively enhance the quality and efficacy of the adversarial patches crafted to mislead the gait recognition model. The training process is directed by a specific objective function, which is formulated as follows:

4 $$\begin{aligned} \min _{G} \max _{D} V(D, G) = {\mathbb {E}}{x \sim p{data}(x)}[\log D(x)] + {\mathbb {E}}_{z \sim p_z(z)}[\log (1 - D(G(z)))] \end{aligned}$$

Also, we employ an iterative optimization process in the training of the Generative Adversarial Network (GAN). This process is a core element in our adversarial training strategy, where the generator, *G*, focuses on producing adversarial patches that simulate real gait data, aiming to deceive the deep learning-based gait recognition models. Concurrently, the discriminator, *D*, hones its ability to distinguish between authentic gait data and these synthetically generated patches. This iterative feedback mechanism between *G* and *D* is crucial, as it allows for the continuous refinement of the adversarial patches.

The GAN architecture presented in this work represents a specialized approach to generating adversarial patches that are finely tuned for compromising our gait recognition systems. The intricate design of the generator and discriminator networks, coupled with the adversarial patch training procedure, ensures the creation of highly deceptive patches.

### Adversarial attack utilizing proximal policy optimization

In our innovative approach to evaluate and enhance the robustness of gait recognition models against adversarial attacks, we integrate the advanced capabilities of Proximal Policy Optimization (PPO) with the creative versatility of Generative Adversarial Networks (GANs). Our strategy revolves around the strategic implementation of adversarial patches, created by GANs, into gait images. These patches are then optimized and positioned using PPO to effectively test the resilience of the deep learning models under adversarial conditions.

PPO, a sophisticated reinforcement learning algorithm developed by Schulman et al. [84], plays a crucial role in our methodology. Unlike traditional policy optimization methods, PPO is designed to adaptively adjust its strategy, ensuring a balance between performance improvement and policy stability. This balance is critical in preventing drastic policy shifts that could undermine the attack’s effectiveness.

In the context of our research, PPO is employed to determine the most impactful placement of adversarial patches within Gait Energy Images (GEIs). The patches, generated by the GAN framework, are placed in various positions and orientations within the GEIs. PPO guides this placement process, evaluating and optimizing the position and orientation of each patch to maximize the potential for misclassification by the CNN model.

**Fig. 6:**
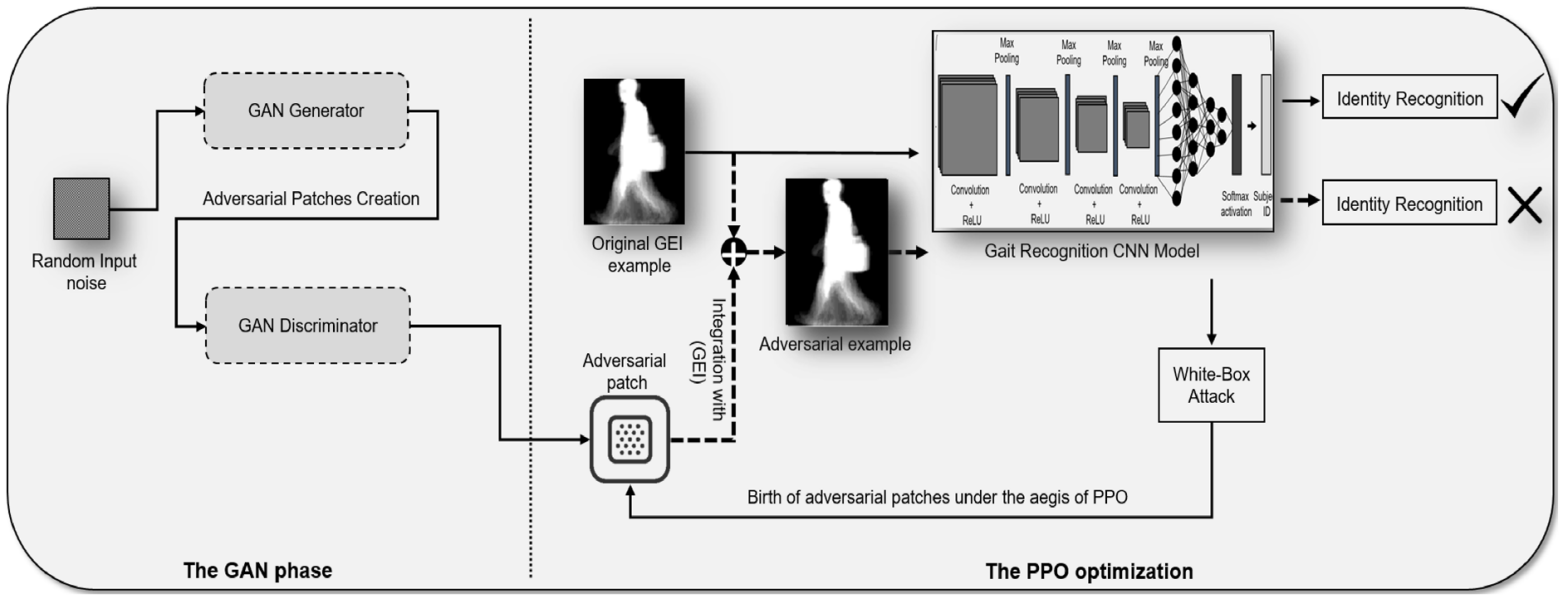
Diagrammatic Overview of the PPO-driven Adversarial Attack Workflow. This comprehensive diagram illustrates the full adversarial attack pipeline. In the GAN phase, the Generator crafts adversarial patches, which are refined through the adversarial feedback loop with the Discriminator. The generated patches are then applied to original GEI examples, creating adversarial examples. The PPO optimization phase ensures strategic placement of these patches, manipulating the gait recognition CNN model in a white-box attack scenario. This intricate process simulates how adversarial patches can mislead the model into incorrect identity recognition.

Our approach, while sharing some similarities with previous efforts that utilized Q-learning, represents a significant advancement due to the incorporation of PPO. This integration leads to a more nuanced and effective adversarial attack, capable of revealing subtle vulnerabilities in the gait recognition system. The overall process, from the generation of patches by GANs to their strategic placement and evaluation using PPO, is illustrated in Fig. 6. This diagram provides a comprehensive overview of the PPO-driven adversarial attack workflow, demonstrating the seamless interplay between patch generation, optimization, and their impact on the performance of the gait recognition system.

#### Optimizing adversarial patch placement

At the core of our approach is the implementation of an adversarial patch, pivotal in challenging the accuracy of our gait-based recognition model. The target model, denoted as *f*, processes Gait Energy Images (GEIs) with dimensions $240 \times 240 \times 1$. Under standard conditions, this model outputs the class label *t*, accurately identifying the gait pattern represented in the GEI.

Our method introduces an adversarial patch, $v = (x, y)$, which is an $n \times n$ matrix composed of pixel values ranging from 0 to 255. This patch is not placed arbitrarily; instead, we harness the Proximal Policy Optimization (PPO) algorithm for its strategic placement on the GEI.

The PPO algorithm, a standout in the realm of reinforcement learning, iteratively explores the GEI, evaluating numerous potential placements for the patch *v*. Each position is assessed based on its effect on the model’s output. The algorithm’s objective is to discover a location where adding the patch ($f(x + v)$) deviates the model’s classification from the correct label *t*. This deviation is indicative of a successful adversarial attack, leading to a misclassification by the model. Crucial to our method is the balance between the patch’s potency and its inconspicuousness. The patch must be discreet enough to elude human detection while potent enough to deceive the CNN model. This balance is achieved by choosing the pixel values and dimensions of the patch to ensure it subtly blends into the GEI, providing a significant yet visually unobtrusive modification.

In the technical execution of PPO, we employ an episodic approach where each episode consists of selecting a potential patch location and evaluating its impact on the model’s output. The reward mechanism within PPO is designed to encourage placements that maximize misclassification while maintaining the patch’s stealth. This is quantified by a reward function that assigns higher scores to patch placements leading to greater deviations from the correct classification. Moreover, the PPO algorithm leverages a policy network that iteratively updates its parameters based on the observed rewards. This network, structured with multiple layers and non-linear activations, efficiently navigates the high-dimensional space of possible patch placements. The policy network’s objective is to converge to an optimal policy that consistently identifies the most effective patch locations. Through this application of PPO, our adversarial attack method achieves a nuanced exploration of the GEI space, pinpointing locations where adversarial manipulations yield the highest impact.

#### Proximal policy optimization

In our adversarial attack framework, we employ Proximal Policy Optimization (PPO) to refine the placement of adversarial patches generated by our GAN. PPO, a state-of-the-art reinforcement learning technique, is utilized to strategically position these patches on Gait Energy Images (GEIs), thereby challenging the robustness of our gait recognition model.

Our implementation of PPO is designed for white-box attacks, where complete knowledge about the target model’s architecture and parameters is available. This allows for finely-tuned adversarial strategies to be crafted, exploiting specific vulnerabilities of the model. PPO, as a reinforcement learning algorithm, navigates through a Markov decision process (MDP), characterized by the tuple $(S, A, P, R, \gamma )$. Here, *S* represents the set of all potential pixel locations on the GEI for adversarial patch placement, *A* denotes the actions related to patch manipulation, *P* is the transition probability, *R* is the reward function guiding effective patch placement, and $\gamma$ is the discount factor in the reward calculation.

The PPO algorithm in our study focuses on optimizing the policy $\pi (s|a)$, which determines the action $a \in A$ in a given state $s \in S$ to maximize the total expected reward, calculated as:

5 $$\begin{aligned} \max E[R(\tau )] \end{aligned}$$

where $R(\tau ) = \sum _{t=0}^{\tau } \gamma ^t r(a_t, s_t)$. However, the policy updates are modified by the inclusion of a surrogate objective function *L*, which is defined as:

6 $$\begin{aligned} L(\theta ) = E_t \left[ \min \left( r_t(\theta ) A_t, \text {clip}(r_t(\theta ), 1-\epsilon , 1+\epsilon ) A_t \right) \right] \end{aligned}$$

where $\theta$ is the policy parameter, $A_t$ is the advantage function at time *t*, $r_t(\theta )$ is the ratio of the new and old policies, and $\epsilon$ is a hyperparameter (usually set to 0.2) that determines how much the new policy can deviate from the old policy.

##### Detailed PPO mechanics

To delve deeper into the Proximal Policy Optimization (PPO) process employed in our study, we focus on its mechanics and how it interacts with the generated adversarial patches for effective placement.

**Fig. 7:**
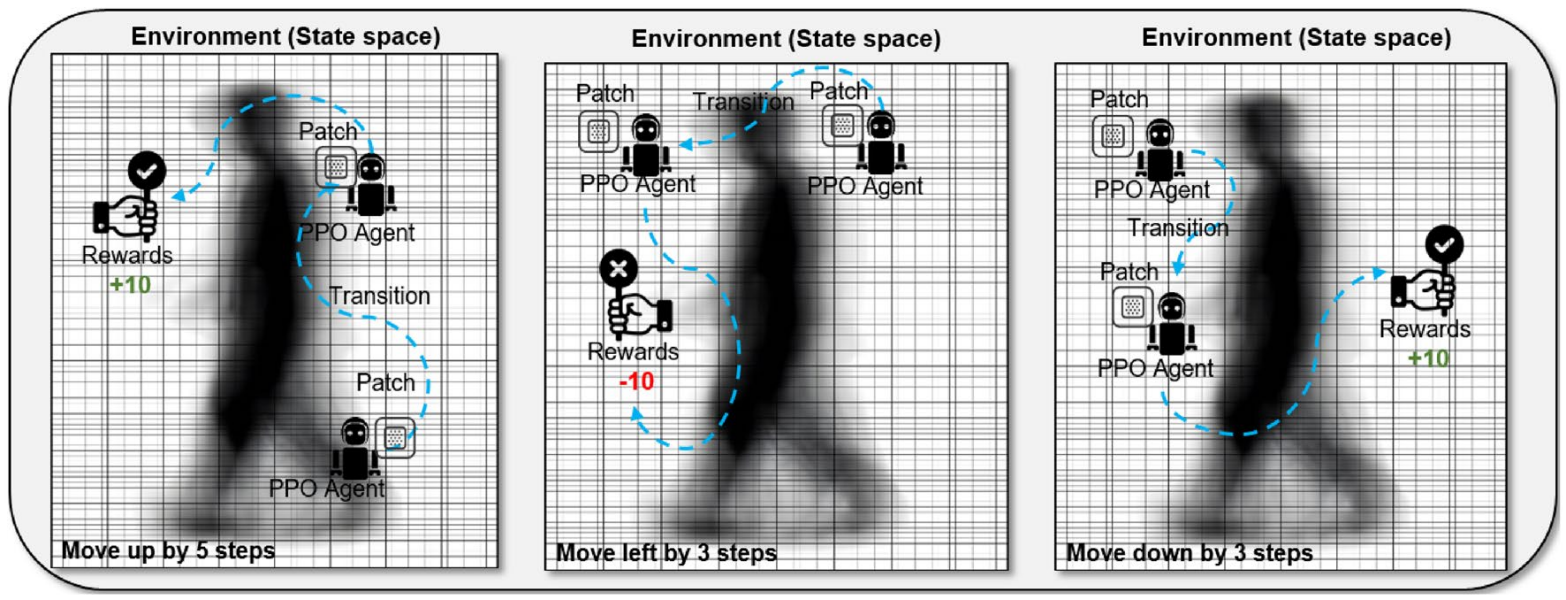
Visualization of the agent’s trajectory in applying adversarial patches and the resulting decision-making process.

- *Modeling* In our PPO-based approach, the agent operates within the two-dimensional environment of a subject’s Gait Energy Image (GEI). This environment is conceptualized as a grid, with each pixel location representing a distinct state. The agent’s task is to interact with this environment by strategically altering pixel values, thereby simulating the process of creating and placing an adversarial patch. This interaction is crucial for determining the most effective placement of the patch to achieve the desired outcome of model misclassification.
- *State space* The state space includes the set of possible states (locations) in which the agent can explore and exploit the environment. In the context of the proposed problem, the states are pixel locations in the GEI of a particular subject. The agent can move to any pixel location to manipulate it and craft the adversarial samples.
- *Actions* At each state, the agent has a set of four possible actions: moving ’up’, ’down’, ’left’, or ’right’ within the GEI grid. These actions allow the agent to traverse the image and explore different patch placements. The choice of action at each state is determined by the policy $\pi (s|a)$, which evaluates the potential effectiveness of each action based on the current state.
- *Agent goals and rewards* Similar to the previous section, rewards are the numerical values assigned to an agent when it takes action in the environment. This reward indicates the quality of an action that the agent takes. We assign two reward values for this problem, +10 and -10. When the agent adds an adversarial patch at a particular location, the quality of this action can be computed by predicting the label of the GEI after adding the patch-based adversarial perturbation. The rewards are defined as follows: if $\rho = f(x) \le p$, reward = 10, otherwise reward = -10. When the agent takes an action to perturb the GEI location, the action quality is determined by computing the confidence *p*, of the model toward the actual class and comparing it with confidence $\rho$ after the perturbation is added. When the agent reaches a location that leads the model/system to misclassify an image when perturbed with an adversarial patch, the episode for an agent is terminated, and the algorithm stops. The placement and impact of these adversarial patches can be visualized in Fig. 7, which illustrates the agent’s trajectory and decision points throughout the process.

#### Implementing the algorithm

The Proximal Policy Optimization (PPO) algorithm is implemented in our adversarial attack framework, following a systematic process to determine the optimal location for the adversarial patch. The key steps of this process are outlined as follows:

- Initialization: The policy parameters $\theta$ and the old policy parameter $\theta _{\text {old}}$ are initialized. This sets the baseline for the learning process of the PPO agent.
- Trajectory Collection: We collect a set of trajectories $D = {\tau _i}$ by executing the policy $\pi _{\theta }$ within the simulated environment. These trajectories comprise the sequences of states, actions, and rewards encountered by the agent.
- Reward Computation: The ’rewards-to-go’ $R_t$ are calculated. These represent the sum of future rewards expected from a particular state onwards, providing a foresight into the potential outcomes of actions taken.
- Advantage Estimation: We compute advantage estimates $A_t$, which are crucial for assessing the relative benefit of each action compared to the average at a given state.
- Policy Update: The policy undergoes an update process by maximizing the PPO-Clip objective via stochastic gradient ascent, typically employing the Adam optimizer. This step is pivotal in refining the policy to yield better results.
- Iteration: This entire process is iteratively conducted with the updated policy parameters. Each iteration contributes to the continuous learning and adaptation of the agent, enhancing its ability to pinpoint the most effective location for adversarial patch placement.

The implementation of PPO in our adversarial attack approach is encapsulated in Algorithm 1, which details the procedural steps taken by the PPO agent in the environment.

**Algorithm 1:**
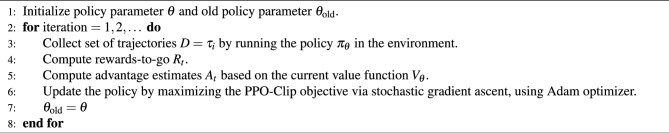
Proximal Policy Optimization for Adversarial Attacks

This comprehensive approach ensures that the adversarial patch is strategically placed to exert maximum impact on the gait recognition model.

#### Adversarial patch placement and impact

The outcome of the PPO-driven approach is the strategic placement of adversarial patches within the GEI. The precise placement of these patches is crucial, as it determines the effectiveness of the attack. We consider various patch transition steps (1, 3, 5, and 10 steps) to evaluate the impact of different levels of patch manipulation. The entire process, including the decision-making trajectory of the agent and the resulting impact on the model’s classification accuracy, is illustrated in Fig. 7.

## Experimental results and analysis

This section presents the empirical results from our proposed adversarial attack methodology, organized as follows:

- Baseline Performance Evaluation: We start by evaluating the performance of our deep learning-based gait recognition system in a standard scenario, without any adversarial interference. This evaluation establishes a benchmark for understanding the system’s functionality under normal conditions.
- Impact of Adversarial Attacks: After setting the baseline, we analyze the system’s performance when subjected to adversarial attacks. This section aims to identify the system’s vulnerabilities and assess its robustness against such attacks,

### Baseline performance evaluation

To maintain a systematic approach in our evaluation, we begin by understanding the inherent performance of our deep learning-based gait recognition system. This assessment, executed in a controlled environment without any adversarial interference, sets the benchmark against which we will compare the effects of the adversarial attacks.

#### Experimental setup

Our experimental setup is meticulously crafted to capitalize on the distinct characteristics of both the CASIA Gait Database: Dataset B and the OU-ISIR Treadmill dataset B -Clothes variation-, offering a comprehensive evaluation platform for our gait recognition approach. For CASIA-B, we followed the protocol by Wu et al^46^ and Zhang et al^47^, using the first 74 subjects for training and the remaining 50 for testing to cater to conditions like NM, BG, and CL. We carefully curated the training set with the initial sequences of these 74 subjects, while the subsequent sequences from the remaining 50 provided a diverse test bed.

For the OU-ISIR dataset, we adhered to the developers’ guidelines^48^, forming a training set with 20 subjects in varied clothing for robust model training, a gallery set of 48 subjects in consistent attire for recognition, and a probe set with these 48 subjects in alternate clothing to test verification strength. This strategic subdivision facilitates an equitable and rigorous evaluation, mirroring real-world application challenges.

Preprocessing and data normalization, as detailed in 3.2, were integral to our approach, ensuring data uniformity and optimizing input quality. The CASIA-B dataset’s real-world variance, alongside OU-ISIR’s controlled settings, enables a thorough assessment of our method’s resilience and precision across diverse scenarios, demonstrating its robustness and adaptability.

#### Metrics for baseline evaluation

In the specialized domain of gait recognition, accurately evaluating the effectiveness and reliability of our system is essential. This evaluation depends on carefully selected performance metrics that are designed for gait analysis. These metrics are critical not only for measuring the accuracy and efficiency of our system but also for identifying its strengths and weaknesses in various scenarios. In our research, we have adopted the following key metrics, which are widely recognized and highly relevant in the field of gait recognition research :

1. *Accuracy (Acc)* A fundamental measure in recognition domains, accuracy underscores the system’s aptitude in correctly discerning and categorizing gaits. It is defined as:
  7 $$\begin{aligned} \text {Acc} = \frac{\text {Number of Correct Identifications}}{\text {Total Recognition Attempts}} \end{aligned}$$
  An elevated accuracy typically serves as a testament to the system’s robustness within the given evaluation parameters.
2. *Precision* A measure of the system’s accuracy in making positive identifications, precision quantifies the proportion of correct positive identifications among all positive identifications made. It is calculated as:
  8 $$\begin{aligned} \text {Precision} = \frac{\text {True Positives}}{\text {True Positives} + \text {False Positives}} \end{aligned}$$
  High precision reflects the system’s effectiveness in minimizing false positive errors.
3. *Recall* Also known as the True Positive Rate, recall measures the system’s ability to correctly identify all legitimate instances. It is expressed as:
  9 $$\begin{aligned} \text {Recall} = \frac{\text {True Positives}}{\text {True Positives} + \text {False Negatives}} \end{aligned}$$
  High recall indicates the system’s proficiency in correctly recognizing genuine users.
4. *F1 Score* The F1 Score is a harmonic mean of precision and recall, providing a singular metric to balance the trade-off between the two. It is defined as:
  10 $$\begin{aligned} F1 = 2 \times \frac{\text {Precision} \times \text {Recall}}{\text {Precision} + \text {Recall}} \end{aligned}$$
  A high F1 Score signifies that the system robustly maintains both high precision and high recall.
5. *False acceptance rate (FAR)* In the domain of gait recognition, which often plays a critical role in security-sensitive applications, FAR is a pivotal metric that measures the rate at which unauthorized individuals are mistakenly identified as legitimate subjects. It is calculated as:
  11 $$\begin{aligned} \text {FAR} = \frac{\text {Number of False Acceptances}}{\text {Total Recognition Attempts}} \end{aligned}$$
  A low FAR is desirable as it reflects the system’s robustness in accurately distinguishing between authorized subjects and impostors, thereby mitigating the risk of security breaches.
6. *False rejection rate (FRR)* FRR is a critical measure in gait recognition systems, reflecting the rate at which legitimate users are incorrectly denied access. This rate is defined as:
  12 $$\begin{aligned} \text {FRR} = \frac{\text {Number of False Rejections}}{\text {Total Recognition Attempts}} \end{aligned}$$
  Striking an optimal balance between security and user convenience, a low FRR ensures that authorized users are granted access without undue hindrance, thus enhancing the user experience while maintaining the integrity of the security framework.

These metrics are consistent with those used in biometric system evaluation, including speaker verification tasks^49^.

#### Baseline evaluation results

Drawing from the experimental setup and the performance metrics delineated earlier, we now present the baseline results of our gait recognition system. These results will serve as the foundation upon which adversarial attacks will be subsequently analyzed

The effectiveness of our gait recognition system is validated through extensive testing on the CASIA-B and OU-ISIR datasets 3.1, producing notable results that highlight its robustness in diverse scenarios. The key performance indicators, including accuracy, precision, recall, and F1-Score, are detailed in Table 3. These metrics provide a foundational understanding of the system’s overall performance.

**Table 3:** Comparative performance metrics for the CASIA and OU-ISIR datasets with different gallery and probe configurations.

Gallery	Probe	Accuracy	Precision	Recall	F1-Score
CASIA Gait Database: Dataset B (124 subjects)					
Normal	Normal	99.06%	99.12%	95.88%	97.99%
Normal	Coats	98.95%	99.48%	95.75%	97.05%
Normal	Bags	98.75%	99.29%	94.92%	97.42%
Normal+Bags	Normal	98.73%	96.80%	90.98%	92.49%
Normal+Bags	Coats	98.21%	96.21%	93.88%	95.35%
Normal+Bags	Bags	97.87%	95.93%	90.65%	95.55%
Normal+Coats	Normal	97.84%	96.21%	91.82%	93.87%
Normal+Coats	Coats	97.04%	95.25%	92.70%	93.42%
Normal+Coats	Bags	96.45%	98.61%	92.22%	95.64%
**Average**		**98.10%**	**97.75%**	**93.20%**	**95.42%**
OU-ISIR dataset B -Clothes variation- (68 subjects)					
Standard	Standard	99.19%	99.16%	93.81%	97.50%
Standard	Varied	98.71%	97.45%	93.19%	96.91%
Standard+Varied	Standard	96.94%	96.06%	92.51%	92.25%
Standard+Varied	Varied	96.12%	95.49%	88.29%	91.06%
**Average**		**97.74%**	**97.04%**	**91.95%**	**94.43%**

**Fig. 8:**
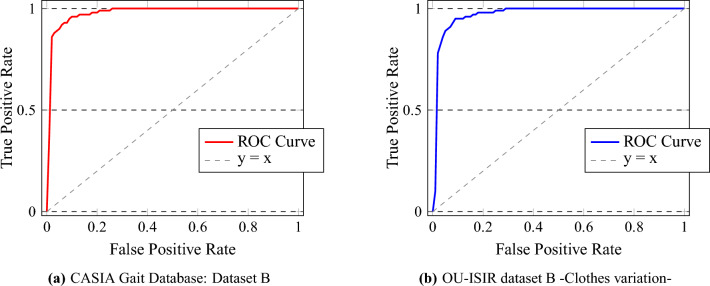
Comparative Analysis of ROC Curves for CASIA Gait Database: Dataset B and OU-ISIR dataset B -Clothes variation-.

To provide a more detailed analysis, Receiver Operating Characteristic (ROC) curves for each dataset are presented in Fig. 8. These curves effectively illustrate the system’s capability to distinguish between true positives and false positives across different threshold levels, highlighting its discriminative power.

**Fig. 9:**
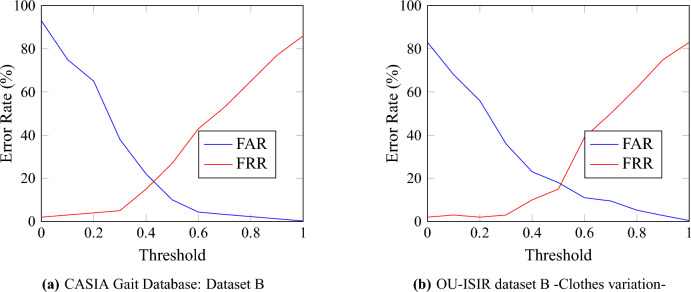
Error Rates for CASIA B and OU-ISIR Datasets.

Additionally, the Error Rate diagrams, depicted in Fig. 9, plot the False Acceptance Rate (FAR) and False Rejection Rate (FRR) for both datasets. These plots reveal the system’s adeptness at striking a critical balance: safeguarding against unauthorized access (minimizing FAR) while ensuring authorized users can use the system smoothly and efficiently (minimizing FRR). Such a balance is paramount in the practical deployment of gait recognition systems, where user experience and security must be jointly optimized.

The comprehensive analysis encompassed by the metrics in the table and the visual data in the figures offers a holistic view of the system’s capabilities, underscoring its potential and reliability in the field of gait recognition.

#### Analysis of baseline evaluation results

The empirical evaluations performed on the CASIA-B and OU-ISIR datasets highlight the effectiveness of our gait recognition system, underscoring its robust computational architecture and recognition capabilities. On the CASIA-B dataset, the system attained a significant accuracy of 98.10%, accompanied by precision and recall rates of 97.75% and 93.20%, respectively, resulting in an F1-score of 95.42%. These metrics demonstrate the system’s capability to accurately identify and classify complex gait patterns across a wide range of walking conditions. The balanced trade-off between precision and recall highlights the system’s calibrated sensitivity to accurate gait patterns, effectively minimizing both overgeneralization and overfitting. Exploring the details of the OU-ISIR dataset’s results, which showcase the system’s resilience against gait variations induced by different clothing, we note a commendable accuracy of 97.74%. Although slightly lower, it is significant given the additional complexity introduced by clothing variation. The precision, recall, and F1-scores, at 97.04%, 91.95%, and 94.43% respectively, further confirm the system’s consistent performance in the face of nuanced gait alterations.

The Receiver Operating Characteristic (ROC) curves, as depicted in Fig. 8, provide a quantitative visualization of our system’s classification efficacy. For the CASIA Gait Database: Dataset B, the ROC curve approaches the upper left corner, indicating a high true positive rate with a minimal false positive rate, a desirable outcome in gait recognition. This is reflected in an Area Under Curve (AUC) value that is close to 1, underscoring excellent performance. Similarly, the ROC curve for the OU-ISIR dataset B with clothes variation demonstrates the system’s resilience to changes in appearance, maintaining a high true positive rate. Despite the potential for clothing to introduce noise into gait data, the system’s discriminative capability is hardly impacted, as evidenced by the ROC curve maintaining proximity to the ideal line. These ROC curves collectively highlight the system’s discriminating power, effectively differentiating between genuine and impostor gait patterns across varied conditions. The AUC metrics, serving as an aggregate measure of performance across all possible classification thresholds, confirm the system’s robustness and the effectiveness of its underlying computational architecture.

Furthermore, the balance between the False Acceptance Rate (FAR) and False Rejection Rate (FRR) observed across both datasets underscores the system’s dual proficiency in security and usability. The achievement of a low False Acceptance Rate (FAR) underscores the system’s capabilities in thwarting unauthorized access, a critical feature for high-security environments. Simultaneously, maintaining moderate levels of False Rejection Rate (FRR) guarantees that legitimate users encounter minimal barriers to access.

In conclusion, the analysis, which includes measures such as accuracy, precision, recall, F1-scores, ROC curves, False Acceptance Rate (FAR), and False Rejection Rate (FRR), decisively demonstrates our system’s readiness to operate efficiently under various real-world conditions. The results validate the system’s optimized balance between accuracy and error rates, positioning it as a solution suitable for deployment in diverse gait recognition applications.

### Impact of adversarial attacks

The subsequent phase of our research focused on evaluating the resilience of our deep learning-based gait recognition system against adversarial attacks. We employed a novel adversarial strategy that combines Proximal Policy Optimization (PPO) with Generative Adversarial Networks (GANs). This hybrid approach enabled us to conduct adversarial attacks on our gait recognition models and rigorously examining their robustness. The execution of these attacks was carried out with strategic precision, subtly altering the recognition process to mirror real-world adversarial conditions. This phase was critical in evaluating the durability of our models against malicious interventions and in understanding their behavior under adversarial circumstances.

In the following sections, we present a detailed analysis of these adversarial attacks. We assess their impact using various performance metrics, providing a comprehensive view of the effects of adversarial interference on gait recognition systems.

#### Metrics for adversarial attack evaluation

The effectiveness of the adversarial attack evaluation hinges on a set of carefully selected metrics. Each metric provides insight into a different facet of the attack’s impact on the Gait Recognition System, collectively offering a comprehensive understanding of its repercussions. The metrics employed in this evaluation are described as follows:

1. *Adversarial Accuracy (AdvAcc)* This metric provides an assessment of the system’s robustness by measuring the rate of correct classifications even when the input data has been manipulated by an adversary. It is a critical indicator of how well the recognition system can resist and withstand the specific manipulations designed to evade or fool it. The Adversarial Accuracy is defined as the ratio of the number of correct predictions to the total number of attempts during adversarial conditions, expressed as a percentage:
  13 $$\begin{aligned} AdvAcc = \frac{\text {Number of Correct Predictions under Attack}}{\text {Total Number of Predictions under Attack}} \times 100 \end{aligned}$$
  A high AdvAcc value suggests that the recognition system is capable of effectively countering adversarial attacks, maintaining a high level of performance in identifying the correct gait patterns despite the adversarial perturbations introduced. Alternatively, a significant decrease in AdvAcc compared to the baseline accuracy would underscore the system’s vulnerabilities and identify potential areas for improvement in securing against adversarial attack.
2. *Attack Success Rate (ASR)* ASR measures the proportion of instances where the adversarial attack led to incorrect classifications, indicating the overall effectiveness of the attack. An elevated ASR value is indicative of the attack’s ability to deceive the target system successfully. It is calculated as follows:
  14 $$\begin{aligned} ASR = \frac{\text {Number of Successful Adversarial Perturbations}}{\text {Total Number of Perturbations Attempted}} \times 100 \end{aligned}$$
  A high ASR suggests that the attack can successfully compromise the system, rendering it unreliable under adversarial conditions.
3. *Confidence Reduction* This metric measures the decrease in the system’s predictive confidence due to adversarial perturbations. It reflects how effectively the attack diminishes the model’s certainty in correctly identifying the target class. The calculation is expressed as :
  15 $$\begin{aligned} \text {Confidence Reduction} = \frac{1}{N} \sum _{i=1}^{N} (C_{\text {original},i} - C_{\text {adversarial},i}) \end{aligned}$$
  where $C_{\text {original},i}$ and $C_{\text {adversarial},i}$ denote the confidence scores before and after the attack, respectively. A notable reduction in confidence suggests a significant impact on the model’s reliability.
4. *Perceptibility* The perceptibility of an adversarial patch is pivotal in contexts where the attack’s visibility to human observers must be minimized. This metric evaluates how discernible the changes are, with lower scores indicating less detectable perturbations:
  16 $$\begin{aligned} \text {Perceptibility} = \frac{1}{N} \sum _{i=1}^{N} ||x_i - x_{i}^{'}||_2 \end{aligned}$$
  Here, $x_i$ is the original sample, and $x_{i}^{'}$ is the sample with the adversarial patch. The goal is to achieve a balance where the attack remains effective without being perceptible.

These metrics, when utilized collectively, offer a nuanced evaluation of the adversarial attack’s success and its practical applicability in real-world scenarios. They effectively uncover the vulnerabilities within the Gait Recognition System and highlight the subtlety and effectiveness of the attack, thereby providing valuable insights into the system’s resilience and areas for potential improvement.

#### Adversarial attack results

In the assessment of our adversarial attack’s robustness, we combined the strengths of Proximal Policy Optimization (PPO) and Generative Adversarial Networks (GANs). We focused on manipulating the Gait Energy Image (GEI) for each subject, considering a range of clothing variations to simulate real-world conditions. The PPO agent, starting with no prior knowledge of the GEI, treated it as an exploratory environment. The effectiveness of the agent’s actions, aimed at inducing misclassification, was quantitatively evaluated based on the model’s response to these manipulated GEIs. The agent received rewards proportional to the level of disruption caused, as explained in Section 3.5. This adversarial methodology involved the PPO agent systematically introducing a perturbative element into the GEI a patch consisting of random grayscale pixel values strategically positioned to deceive the recognition model. Through an iterative process of 50 episodes, we optimized the location and size of this perturbation to evaluate the model’s vulnerabilities.

During the experimental phase, we leveraged the substantial subject pools of the CASIA-B and OU-ISIR datasets, placing a particular emphasis on the importance of clothing variation. The CASIA-B dataset, with its well-structured environment, includes 124 subjects recorded in diverse clothing scenarios: normal, wearing coats, and carrying bags. This variety allowed us to construct a comprehensive range of gallery and probe sets, enabling a thorough examination of the system’s performance under varying conditions of clothing and accessories. We organized these sets to mimic real-world scenarios, such as changes in a subject’s gait due to different outfits, to test the model’s ability to consistently identify individuals. The probe datasets were structured to progressively present escalating levels of difficulty, beginning with a subset of 25 subjects and gradually expanding to encompass the entire subject pool. This approach allowed for a thorough examination of the effectiveness of the adversarial attacks across diverse dataset sizes.

The OU-ISIR dataset, known for its extensive array of clothing variations and a substantial number of frames per sequence, served as an excellent complement to the CASIA-B dataset in assessing the system’s performance. For the gallery set, we used sequences featuring standard clothing, whereas the probe set comprised sequences with various types of clothing. This setup rigorously evaluated the model’s ability to recognize individuals under varied conditions. Similarly, for the OU-ISIR dataset, the evaluation was conducted in two phases: initially with the first 20 subjects to establish a preliminary understanding of the model’s performance, followed by an expanded test on the entire set of 68 subjects to thoroughly assess the robustness of our adversarial attack across a broader spectrum of individual representations. For an in-depth view of the standard and various clothing types used in the OU-ISIR dataset, please refer to Table A1 in the Supplementary Material.

The outcomes of our evaluation of adversarial attacks are presented in Table 4. This table showcases the impact of the adversarial attacks on our gait recognition models, which were trained using both the CASIA-B and OU-ISIR datasets, and subjected to varying adversarial patch transition steps. We report the average success rates of the attacks and the corresponding adversarial accuracy for different sizes of subject groups. These results provide valuable insights into the resilience of the model’s performance under assorted adversarial conditions. The assessment emphasizes the system’s reaction to adversarial interventions, particularly noting the variations in attack success rate and adversarial accuracy at different stages of patch transition.

**Table 4:** Summary of adversarial attack evaluation on CASIA-B and OU-ISIR datasets with additional metrics for different transition steps.

Adversarial Attack Evaluation			
Dataset	Probe Set Size	Avg. Attack Success Rate	Avg. Adversarial Accuracy
Adversarial Patch Transition with 1 Step			
CASIA-B	25 Subjects	23.34%	78.18%
	74 Subjects	27.34%	70.57%
	124 Subjects	29.84%	68.75%
OU-ISIR	20 Subjects	17.34%	81.59%
	68 Subjects	20.66%	78.21%
Adversarial Patch Transition with 3 Steps			
CASIA-B	25 Subjects	28.50%	69.10%
	74 Subjects	41.87%	55.86%
	124 Subjects	44.19%	53.03%
OU-ISIR	20 Subjects	24.67%	73.60%
	68 Subjects	33.50%	68.60%
Adversarial Patch Transition with 5 Steps			
CASIA-B	25 Subjects	57.49%	39.98%
	74 Subjects	68.27%	34.62%
	124 Subjects	**65.87%**	**31.40%**
OU-ISIR	20 Subjects	51.56%	51.85%
	68 Subjects	**63.99%**	**40.39%**
Adversarial Patch Transition with 10 Steps			
CASIA-B	25 Subjects	33.22%	68.17%
	74 Subjects	39.35%	63.32%
	124 Subjects	42.16%	60.86%
OU-ISIR	20 Subjects	24.06%	75.00%
	68 Subjects	36.60%	65.10%

A crucial aspect of evaluating our gait recognition system’s resilience against adversarial threats involves observing the variations in confidence levels across different stages of the attack. This confidence metric, reflecting the model’s certainty in its predictions, was systematically documented over a series of iterations (10, 25, 50, and 100), covering the full range of subjects in both the CASIA-B and OU-ISIR datasets. The observed fluctuations in confidence levels serve as indicators of the adversarial attack’s impact, with notable shifts pointing to the strength and effectiveness of the perturbations.

**Fig. 10:**
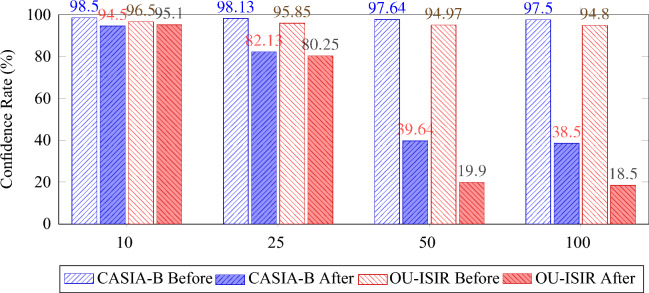
Comparison of Confidence Rate in CASIA-B and OU-ISIR datasets before and after the adversarial attack, illustrating the trends in confidence reduction across 10, 25, 50, and 100 iterations.

The histogram in Fig. 10 visualizes these trends, presenting a comparative view of the confidence rates for the CASIA-B and OU-ISIR datasets across the specified iteration phases. This visual representation reinforces the analytical insights, offering a tangible depiction of the confidence level shifts throughout the adversarial attack process.

**Fig. 11:**
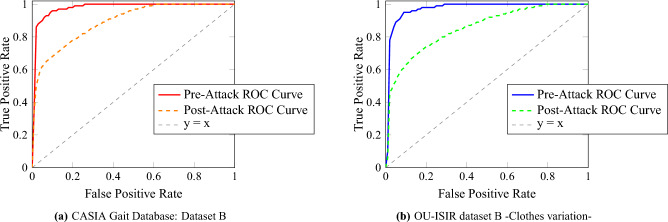
Comparative Analysis of ROC Curves for CASIA Gait Database: Dataset B and OU-ISIR dataset B -Clothes variation-.

The subjection of our gait recognition system to adversarial attacks provided an opportunity to critically evaluate its discriminative performance. Utilizing Receiver Operating Characteristic (ROC) analysis, we could observe the impact of adversarial interventions on the system’s capability to distinguish between authentic and adversarial inputs. This analysis was instrumental in visualizing the true positive and false positive rates across different threshold settings, thereby offering a comprehensive view of the system’s performance post-attack. In Fig. 11, we illustrate the Receiver Operating Characteristic (ROC) curves corresponding to the performance of our gait recognition system, both prior to and following the adversarial attacks.

#### Analysis of adversarial attack results

The experimental results, as tabulated in Table 4, provide a comprehensive evaluation of the adversarial attacks on gait recognition systems trained on CASIA-B and OU-ISIR datasets. This analysis offers a critical assessment of the robustness of these systems under various adversarial scenarios, delineated by different adversarial patch transition steps.

The study reveals a marked decline in the performance of gait recognition models with increased adversarial intervention. This trend is particularly evident in the progressive rise in attack success rates and the corresponding decrease in adversarial accuracy with escalating transition steps. Notably, the peak efficacy of adversarial attacks is observed at 5 steps, where the attack success rates reach their zenith, indicating a heightened vulnerability of the models to this level of adversarial complexity. However, a counterintuitive observation emerges at 10 steps, where the attack efficacy attenuates, as evidenced by a reduced success rate compared to the 5-step scenario. This reduction could be indicative of an overfitting phenomenon, where the adversarial patches, excessively tailored to the model, lose their effectiveness in generalizing across varied gait samples. Alternatively, it may suggest the existence of a complexity threshold in adversarial strategies, beyond which further modifications cease to proportionally escalate the attack’s impact. This nuanced finding underscores the necessity of a calibrated approach in the design of adversarial attacks, where the complexity is optimized to achieve maximum disruption. The optimal results at 5 steps, in particular, highlight the significance of identifying the most effective level of adversarial intervention.

The incorporation of transition steps as a pivotal experimental variable provides significant insights into the models’ resilience against incremental adversarial modifications. In the context of the CASIA-B dataset, we observed a substantial increase in attack success rates with the escalation of transition steps, reaching a peak impact at 5 steps. This pronounced rise in vulnerability at an intermediate level of adversarial complexity suggests an optimal point of attack efficacy, beyond which, particularly at 10 steps, the effectiveness of the attack starts to plateau or even decrease. This phenomenon points to a nuanced balance between the complexity of adversarial interventions and their effectiveness, highlighting a potential threshold in adversarial strategy efficacy. Contrastingly, the OU-ISIR dataset exhibits a different response pattern, with a generally subdued sensitivity to adversarial modifications compared to CASIA-B. This variation in response could be attributed to the inherent characteristics of the OU-ISIR dataset, such as the diversity of subjects, the nature of gait patterns, and specific environmental conditions of data capture. These findings suggest that the OU-ISIR dataset may possess certain inherent defenses against adversarial attacks, although not absolute. The contrasting responses of the two datasets to the same adversarial tactics underscore the significance of dataset-specific attributes in influencing model robustness and vulnerability.

This analysis underscores the need for a tailored approach to adversarial defense, taking into account the unique challenges and attributes presented by different datasets. The observed optimal effectiveness at 5 transition steps in the CASIA-B dataset, in particular, opens new avenues for exploring the dynamic interplay between adversarial patch complexity and model susceptibility. Understanding these aspects is crucial for developing more sophisticated and effective adversarial defense mechanisms, ensuring that gait recognition systems are resilient against a broad spectrum of adversarial threats.

The analysis of attack success rates offers pivotal insights into the differential impact of adversarial strategies on the CASIA-B and OU-ISIR datasets. In the CASIA-B dataset, the escalation of success rates with increasing transition steps is particularly noteworthy. This trend not only reflects the model’s heightened vulnerability to more sophisticated adversarial techniques but also signals a potential gap in the existing defensive frameworks. The pronounced peak in success rates observed at 5 steps, followed by a slight dip at 10 steps, suggests an optimal threshold of adversarial complexity that maximizes impact. This nuanced pattern of response underscores the need for dynamic and adaptable defense strategies, capable of countering varying levels of adversarial complexity.

In contrast, the OU-ISIR dataset presents a different narrative. While it also exhibits an increase in attack success rates with more transition steps, the overall rates are lower compared to CASIA-B. This indicates a relative resilience inherent in the OU-ISIR dataset, potentially stemming from its diverse clothing variations and distinct gait patterns. However, the fact that the success rates do increase, albeit at a slower pace, points to the inevitability of vulnerabilities even in seemingly robust models. It suggests that while the OU-ISIR dataset may possess certain defensive advantages, it is not immune to sophisticated adversarial attacks, particularly those that are fine-tuned and incrementally escalated.

The divergent patterns observed in the two datasets highlight the complex landscape of adversarial threats in gait recognition. They emphasize the importance of a multifaceted approach to defense, one that is not only responsive to the level of adversarial complexity but also attuned to the specific characteristics and vulnerabilities of different datasets. This approach should integrate proactive defense mechanisms that evolve in tandem with advancing adversarial tactics, ensuring a robust defense posture against a broad spectrum of potential threats. Furthermore, these findings advocate for ongoing research to dissect and understand the unique aspects of each dataset, leveraging such insights to bolster the resilience of gait recognition models in a continually evolving adversarial environment.

In the histogram presented in Fig. 10, Our analysis unfolds in a step-wise manner, mapping out the trajectory of confidence erosion at key intervals. At the outset, a gradual decrease in confidence levels is noted, indicating an initial impact of the adversarial interference. By the 25th iteration, a marked acceleration in the reduction of confidence levels is observed, suggesting a heightened vulnerability of the model under sustained adversarial influence. This trend continues into the 50th iteration, with a notable yet modulating decrease in confidence levels, hinting at an evolving response of the model to the adversarial stimuli. The analysis takes a nuanced turn as it extends to the 100th iteration. Here, the rate of confidence reduction exhibits a different pattern, implying a possible adaptation or an equilibrium state in the model’s reaction to prolonged adversarial presence. Such observations are pivotal in understanding the temporal aspects and the progression of adversarial attacks on gait recognition systems.

Upon examining Fig. 11, we can observe that, the ROC curves for both CASIA-B and OU-ISIR datasets underwent significant transformations. Initially, for CASIA-B, the system displayed a high true positive rate with minimal false positives, indicating a robust discriminative ability. However, post-attack, the ROC curve shifted closer to the diagonal line, reflecting a notable decline in the system’s ability to accurately classify gait samples. The OU-ISIR dataset exhibited a similar trend. While initially demonstrating a strong capacity to discriminate between true and false positives, the adversarial attack led to a marked performance degradation. The post-attack ROC curve moved discernibly towards the diagonal, signaling a reduction in the system’s discriminative efficiency.

This shift in the ROC curves post-attack for both datasets, visually represented in Fig. 11, is indicative of the significant impact that adversarial modifications can have on the performance of gait recognition systems. The comparative analysis of the ROC curves, before and after the attacks, underscored the need for more resilient adversarial defense mechanisms. The analysis also highlighted the importance of continuously evolving these defense strategies to mitigate the effects of such sophisticated attacks, ensuring the reliability and robustness of gait recognition systems in various application scenarios.

The ROC curves depicted in Fig. 11 reveal the system’s response to adversarial challenges. Pre-attack, the system demonstrates a higher true positive rate for a given false positive rate, as shown by the blue curve. The post-attack red curve, however, indicates a decrease in true positive rates across the spectrum of false positive rates, suggesting a noticeable impact on the system’s discriminative ability due to the adversarial intervention.

The analysis of the gait recognition system’s performance under adversarial conditions, as delineated by the ROC curve comparison, elucidates a clear demarcation between the system’s robustness pre- and post-attack. Prior to adversarial intervention, the system exhibited strong discriminative capabilities, efficiently distinguishing between authentic and inauthentic inputs. Post-attack, however, there is a discernible decline in performance, as evidenced by the lower true positive rates at equivalent false positive rates. This degradation in discriminative efficacy highlights the potency of the adversarial techniques employed and underscores the necessity for enhanced defensive mechanisms within the system.

#### Empirical comparison with state-of-the-art techniques

This section presents a comparative analysis of our proposed method, which combines Generative Adversarial Networks (GANs) with Proximal Policy Optimization (PPO), against existing adversarial attack approaches in the context of gait recognition. We evaluate the methods based on Adversarial Accuracy, a key metric that reflects the effectiveness of adversarial attacks-lower values indicate more successful deception of the gait recognition system, thereby revealing greater vulnerability.

Table 5 highlights the performance of various state-of-the-art adversarial attack strategies. Our GAN+PPO-based method stands out by achieving the lowest Adversarial Accuracy of 31.40%, indicating a higher potential to disrupt the system’s ability to correctly identify individuals under adversarial conditions. This result demonstrates our approach’s superior ability to generate challenging and effective adversarial examples that significantly degrade recognition performance.

**Table 5:** Comparison of Adversarial Accuracy (Adv Acc) for various adversarial methods. A higher Adversarial Accuracy indicates a less effective adversarial manipulation, whereas a lower Adversarial Accuracy signifies greater system vulnerability.

Method	Adversarial Accuracy (%)
Adversarial attack using GANs (2019)^50^	80
Gait spoofing using masterization (2023)^54^	78
Adversarial attacks using accelerometer-based data (2022)^52^	78.9
Temporal sparse adversarial attack using GAN (2023)^33^	67.78
One cycle attack on gait authentication (2021)^51^	57
Reinforcement learning-based Q-learning attack (2023)^53^	47.96
Infrared Adversarial Patch Generation Based on Reinforcement Learning (2024)^55^	37.70
**Adversarial attack using GAN & PPO (Our Method)**	**31.40**

Compared to recent techniques, including the Infrared Adversarial Patch Generation Based on Reinforcement Learning (2024)^55^, which achieved an Adversarial Accuracy of 37.70%, our GAN+PPO method still demonstrates a clear improvement, outperforming it by over 6 percentage points. This margin is particularly significant given the novelty and technological advancement of infrared-based approaches in the field.

To provide further context, the One cycle attack on gait authentication (2021)^51^-previously seen as a key breakthrough-recorded an Adversarial Accuracy of 57%, which is 25.6 percentage points higher than our result. Similarly, other 2023 approaches such as the Q-learning-based attack^53^ fall short with a score of 47.96%, reinforcing the effectiveness of our proposed method.

These findings underscore not only the efficacy of combining GANs with PPO but also the evolving threat landscape in gait recognition. As adversarial techniques become increasingly sophisticated, this analysis reaffirms the urgent need for robust and adaptive defense mechanisms capable of resisting such nuanced attacks.

### Model complexity and runtime evaluation

To evaluate the practicality of our proposed approach, we performed a detailed analysis of the complexity and computational efficiency of the model. The CNN-based gait recognition model contains approximately 5.1 million parameters, while the GAN architecture, including both generator and discriminator networks, comprises about 3.4 million parameters.

All experiments were performed using a single NVIDIA RTX 3090 GPU with 24GB of VRAM. The average processing time per batch (32 Gait Energy Images) was 0.84 seconds during training and 0.23 seconds during inference for the CNN model. For adversarial patch generation and placement using PPO, each episode (50 steps) took approximately 7.2 seconds, including the PPO optimization loop.

These results highlight the feasibility of applying our method in research and evaluation scenarios with access to a standard high-end GPU, supporting its deployment for vulnerability assessment of gait recognition systems.

## Conclusion

Our research delved into the resilience of deep learning-based gait recognition systems, particularly focusing on their vulnerability to adversarial attacks. Through the integration of Proximal Policy Optimization and Generative Adversarial Networks, we have unveiled critical insights into how adversarial attacks can exploit and challenge these systems. Our findings highlight the susceptibility of gait recognition models to adversarial methods, emphasizing the need for more robust defense mechanisms in these systems.

This study contributes a novel perspective to the field of gait recognition by intertwining it with deep learning and the complexities of adversarial attacks. The significance of our research is particularly pronounced in the context of increasing reliance on gait recognition for surveillance and identity verification purposes. It underlines the necessity for future gait recognition technologies to incorporate sophisticated adversarial defenses to keep pace with the evolving nature of cyber threats.

The practical implications of our findings are particularly relevant in security-sensitive environments where gait recognition is employed. The study advocates for the design of future gait recognition systems that are fortified against adversarial attacks, thereby ensuring their reliability and integrity in real-world scenarios.

While our investigation provides extensive insights, it also recognizes certain limitations, such as the focus on specific datasets and models. Future research can extend our work by exploring diverse datasets, various deep learning architectures, and different adversarial attack methodologies. Such expansive research will offer a broader understanding of the resilience of gait recognition systems against adversarial threats.

In conclusion, this paper not only exposes the challenges that adversarial attacks pose in gait recognition but also accentuates the critical need for developing more secure and dependable recognition systems. As artificial intelligence continues to integrate into essential applications, the imperative to reinforce these technologies against emerging cyber threats grows. Our study calls for continued research and innovation in this field, aiming to bolster the security and effectiveness of gait recognition technologies in an increasingly digital world.

## Supplementary Information

Supplementary Information.

## Data Availability

The research data used in this study are publicly available. The CASIA Gait Database: Dataset B can be accessed at http://www.cbsr.ia.ac.cn/english/GaitAccess to these datasets may require permission from the respective institutions.
