# Supplementary material for: Evaluating gait system vulnerabilities through PPO and GAN-generated adversarial attacks
Source: Sci Rep. 2026 Jan 23;16:6039. doi: 10.1038/s41598-026-37011-1 (PMC12901303; doi:10.1038/s41598-026-37011-1)
Supplement: Supplementary file 1 — Supplementary Information. [file 41598_2026_37011_MOESM1_ESM.pdf]

Appendix

The OU-ISIR dataset, characterized by its extensive clothing variations, was a vital component of our study, especially in evaluating the robustness of the gait recognition system against adversarial attacks in realistic scenarios. This dataset's detailed composition of clothing types, which is pivotal for our experiments, is systematically listed in **Supplementary Table 1**.

To enhance the clarity regarding the application of this dataset within our research, **Supplementary Table 1** is divided into two columns: Standard Clothing and Diverse Clothing Styles. The 'Standard Clothing' column lists the attire used for the gallery set images, representing the model's baseline learning phase. The 'Diverse Clothing Styles' column enumerates the varied clothing configurations applied to the probe set images, demonstrating the complexity of conditions under which the model's recognition capabilities were tested.

**Standard Clothing**	**Diverse Clothing Styles**
Regular Pants & Half Shirt	Regular Pants & Half Shirt & Hat
Casual Pants & Half Shirt	Regular Pants & Half Shirt & Casquette Cap
Casual Pants & Full Shirt	Regular Pants & Long Coat
Casual Wear	Regular Pants & Long Coat & Muffler
Full Shirt	Regular Pants & Long Coat & Hat
	Regular Pants & Long Coat & Casquette Cap
	Regular Pants & Full Shirt
	Regular Pants & Parka
	Regular Pants & Down Jacket
	Regular Pants & Down Jacket & Muffler
	Casual Pants & Long Coat
	Casual Pants & Parka
	Casual Pants & Down Jacket
	Baggy Pants & Half Shirt
	Baggy Pants & Long Coat
	Baggy Pants & Full Shirt
	Baggy Pants & Parka
	Baggy Pants & Down Jacket
	Short Pants & Half Shirt
	Short Pants & Parka
	Rain Coat & Rain Coat
	Skirt & Half Shirt
	Skirt & Full Shirt
	Skirt & Parka
	Skirt & Down Jacket
	Regular Pants & Full Shirt & Hat
	Regular Pants & Full Shirt & Casquette Cap
	Short Pants & Full Shirt

Table 1: Supplementary Table 1 – Detailed Composition of Clothing Types in the OU-ISIR Dataset: This table enumerates the standard and diverse clothing styles within the OU-ISIR dataset, reflecting the experimental conditions under which the gait recognition system's robustness was evaluated.
